# “I can make more from selling medicine when breaking the rules” – understanding the antibiotic supply network in a rural community in Viet Nam

**DOI:** 10.1186/s12889-019-7812-z

**Published:** 2019-11-26

**Authors:** Hong Hanh Nguyen, Dang Phuc Ho, Thi Lan Huong Vu, Khanh Toan Tran, Thanh Do Tran, Thi Kim Chuc Nguyen, H. Rogier van Doorn, Behzad Nadjm, John Kinsman, Heiman Wertheim

**Affiliations:** 1 Hanoi Medical University, Dong Da, Ha Noi, Vietnam; 2 Wellcome Trust Asia Programme - Oxford University Clinical Research Unit, Ha Noi, Vietnam; 3 Epidemiology and Global Health Unit, Umeå University, Umeå, Sweden; 4 Department of Public Health Sciences, Global Health (IHCAR), Karolinska Institutet, Stockholm, Sweden; 5 Radboud UMC, Nijmegen, the Netherlands

**Keywords:** Antibiotic access, Antibiotic use, Pharmaceutical suppliers, Vietnam, Low and middle income countries

## Abstract

**Background:**

As in many other low and middle income countries (LIMCs), Vietnam has experienced a major growth in the pharmaceutical industry, with large numbers of pharmacies and drug stores, and increasing drug expenditure per capita over the past decade. Despite regulatory frameworks that have been introduced to control the dispensing and use of prescription-only drugs, including antibiotics, compliance has been reported to be strikingly low particularly in rural parts of Vietnam. This qualitative study aimed to understand antibiotic access and use practices in the community from both supplier and consumer perspectives in order to support the identification and development of future interventions.

**Methods:**

This qualitative study was part of a project on community antibiotic access and use (ABACUS) in six LMICs. The focus was Ba Vi district of Hanoi capital city, where we conducted 16 indepth interviews (IDIs) with drug suppliers, and 16 IDIs and 6 focus group discussions (FGDs) with community members. Drug suppliers were sampled based on mapping of all informal and formal antibiotic purchase or dispensing points in the study area. Community members were identified through local networks and relationships with the field collaborators. All IDIs and FGDs were audio-taped, transcribed and analysed using content analysis.

**Results:**

We identified a large number of antibiotic suppliers in the locality with widespread infringements of regulatory requirements. Established reciprocal relationships between suppliers and consumers in drug transactions were noted, as was the consumers’ trust in the knowledge and services provided by the suppliers. In addition, antibiotic use has become a habitual choice in most illness conditions, driven by both suppliers and consumers.

**Conclusions:**

This study presents an analysis of the practices of antibiotic access and use in a rural Vietnamese setting. It highlights the interactions between antibiotic suppliers and consumers in the community and identifies possible targets for interventions.

## Background

The introduction of antibiotics has been pivotal in extending human life expectancy and preventing millions of deaths from common infections, while also facilitating medical advances in surgery, transplantation and chemotherapy. However, the overuse and misuse of antibiotics has contributed to the emergence and transmission of antibiotic resistant bacteria that may lead us into a post-antibiotic era, through lowering the therapeutic effect of antibiotics. Antibiotic resistance is a major concern in all settings worldwide; by 2050, drug resistant infections are projected to cause 10 million deaths annually in the absence of any intervention [1]. Overall, infections caused by drug resistant bacteria can lead to a two-fold increase in the impact of negative outcomes, including death [2].

Vietnam is one of the major growth centres in Asia for the pharmaceutical industry and this growth is expected to continue over the next 20 years [3]. The country has undergone major policy shifts and economic reforms, including a number of radical changes in the pharmaceutical sector and the introduction of laws and regulations from the government to adapt to this growth (Table 1). There were an estimated 40,000 retail medicine outlets in the country between 2011 and 2013 [3], of which the private sector constituted 30% [4]. Consumers now have more choices for health services [5], and they can obtain medicines through self-medication or private pharmacies [6, 7]. Annual drug expenditure per capita in Vietnam has increased from below 10 USD in 2005 to 44 USD in 2015, and is expected to quadruple to 163 USD in 2025 [3].

**Table 1:** Summary of policy and legal framework relevant for antibiotic supply and dispensing in Vietnam

Timeline	Event	Aspects relevant to antibiotic supply and dispensing
1986	“Đổi Mới” [Renovation] Policy	An economic reform initiated in Vietnam in 1986 with the goal to transform the country from a planned economy to a market economy. Among the most important health sector reforms were the introduction of user fees for health services at public hospitals, legalization of private practice, establishment of private pharmacies and private clinics, liberalization of the pharmaceutical industry, and deregulation of the retail trade in drugs [27].
1996	National Medicines Policy	This policy was released with two basic aims: (1) to ensure a sufficient supply of good quality drugs at acceptable prices, and (2) to ensure appropriate medicine usage.
2003	Decision 1847/2003/DQ-BYT on prescription-only drugs	Following this document, antibiotics are listed among the types of drugs that need to be prescribed by a health practitioner and must be dispensed with a prescription at registered drug supplier.
2005	Drug Law (34/2005/QH11)	One aim was to improve appropriate antibiotic use through the requirement of dispensing antibiotics only with a prescription. Patients are recommended to comply strictly with the prescription and provide feedback and any side effects to prescribers. Advertisements for prescription-only drugs including antibiotics are also prohibited under this law.
2008	Decision 04/2008/QD-BYT on prescriptions in outpatient treatment	This Decision replaced the previous Decision 1847/2003/DQ-BYT and was implemented with some further adjustments in the Circular 05/2016/TT-BYT issued later in 2016. Only doctors working in legal health facilities (and assistant doctors in remote areas) are allowed to prescribe and only prescribe after a medical examination. This regulation also states that doctors should not prescribe to satisfy patients’ irrational requests. And, a prescription is only valid for five days since the date of prescribing.
2011	Circular 46/2011/TT-BYT on Good Pharmacy Practice (GPP)	GPP requires the pharmacy to have proper facilities, to supply quality healthcare products, to record drug consumption, and not to sell prescription-only drugs without a prescription. A pharmacist should be present at their drugstore ro provide consultation to patients if needed, and responsible for providing health information to their clients.

Regulations require that drug outlets dispense antibiotics to clients only when a prescription made by a formal healthcare professional is shown (Table 1). However, the regulatory and enforcement system has not been able to keep up with the rapid changes in the pharmaceutical network. Selling prescription-only drugs without prescription is common, particularly in private pharmacies [4]. A study conducted in 2010 reported a high proportions of antibiotics being sold without prescription: ~ 90% in both urban and rural pharmacies [8]. In subsequent surveys in the same rural area, 72% of children under 5 years old were reported to have antibiotic treatment for respiratory symptoms [9, 10]. In most cases where antibiotics are used without a prescription, mothers reported having used the drugs following the advice of drug sellers or based on previous prescription [11]. Reasons for inappropriate antibiotic dispensing from a supplier perspective in this study, similar to other settings, included perceived client demand and expectations, lack of knowledge on antibiotics and related regulations, and financial incentives [8]. Factors influencing community members to use drugs without a prescription include perceptions that the illness was not severe, time and convenience, attitudes of staff and insufficiency of drugs at public health facilities, and poor control of prescription-only drugs on the market [11].

In addition, informal drug shops that operate without a license play an important, albeit poorly quantified, role in the drug supply network in Vietnam. In other LMICs, as in Vietnam, governments face enormous challenges in regulating private drug stores. These challenges include infringements of pharmaceutical regulations, including illegal provision of restricted drugs, a failure to obtain proper registration, and ignorance of quality standards [12]. Unregistered drug shops are common in LMICs, and the issues associated with this type of suppliers need further research [13].

In this study, we explore the issues of antibiotic access and use in a Vietnamese community with the aim of providing an in-depth and comprehensive understanding of practices in both formal and informal drug distribution networks. We describe the perspectives from both the suppliers and consumers regarding antibiotic access and use, in order to identify the potential underlying factors and mechanisms shaping inappropriate antibiotic usage, and as a means of providing the foundation for developing future interventions.

## Methods

### Study design and participants

This qualitative study was part of a project on community antibiotic access and use (ABACUS) in six Low and Middle Income Countries (LMICs): South African, Ghana, Mozambique, Thailand, Vietnam, Bangladesh [14], conducted between June 2016 and March 2017 in FilaBavi – a demographic surveillance site located in Ba Vi [15]. This is a rural district, 60 km from Hanoi city center, and currently containing 31 communes (smallest administrative unit). The surveillance site contains households from 28 communes in the district with a total population of approximately 274,000 inhabitants as of 2017. Local people can access health services through a public health system consisting of a district hospital, polyclinics and community health centers or through private providers.

**Fig. 1:**
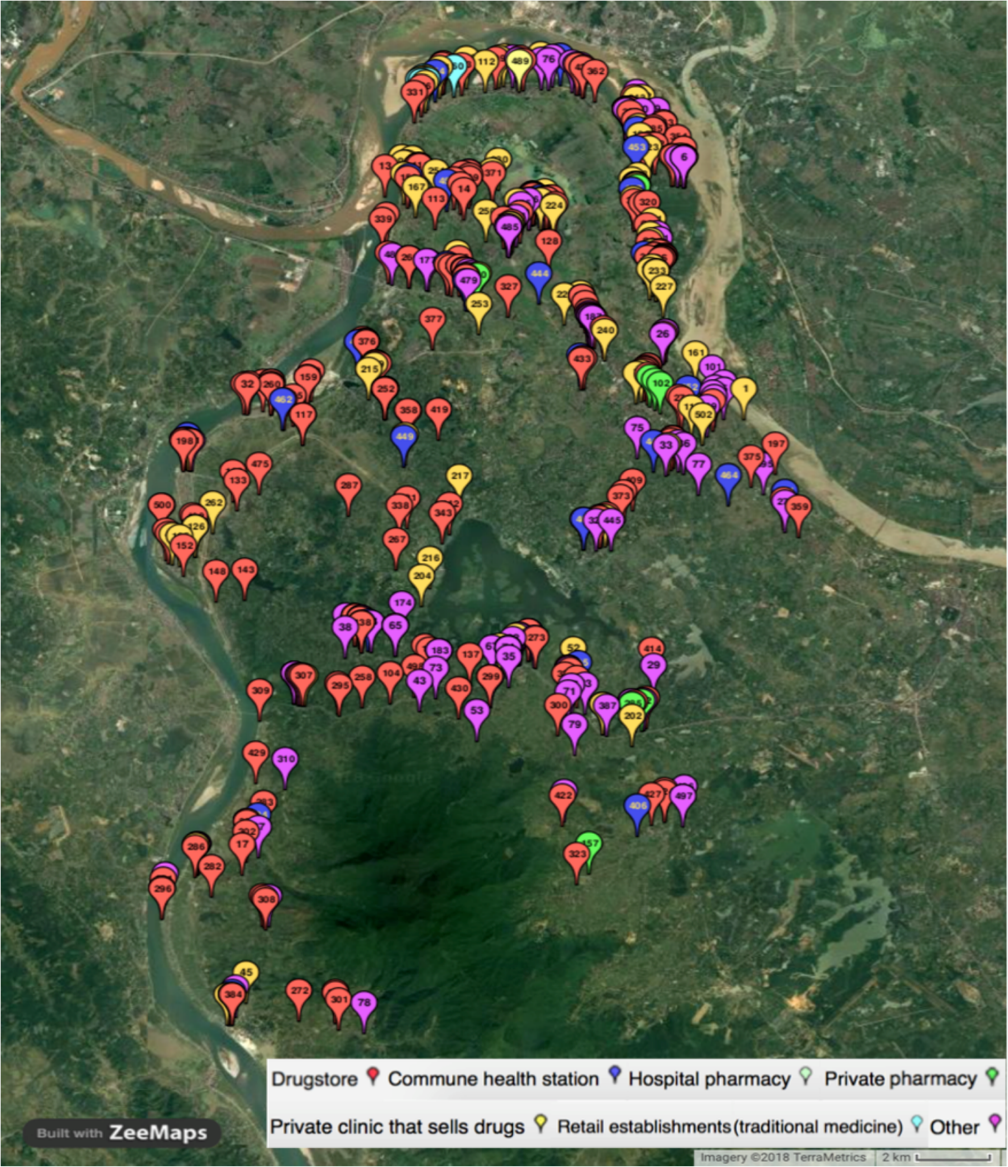
Mapping of antibiotic suppliers in FilaBavi site, Ba Vi district, Hanoi – Viet Nam. Pop-up balloons represent all suppliers identified through the mapping exercise with each color representing one type of suppliers

To establish a basis for sampling and understanding about antibiotic suppliers, we mapped all purchase or dispensing points for antibiotics in the surveillance site using ODK software (University of Washington, Seattle, Washington, USA). Potential dispensing points included all formal or informal antibiotic suppliers (from public hospital pharmacy to street vendor) which were identified based on both official lists provided by local authorities and the knowledge of field collaborators, who were local people residing in the local areas. Altogether, we identified 502 antibiotic suppliers in Ba Vi district (Fig. 1), of which 325 (64.7%) operating informally without a license (Table 2).

**Table 2:** Number of antibiotic suppliers by type and by license status in Fila Bavi identified in ABACUS mapping activity in 2017

Type of antibiotic supplier	Formal	Informal	Total
Public hospital pharmacy	1	0	1
Commune health center pharmacy	28	0	28
Private dispensaries (Drug stores)	109	159	268
Private clinics	7	77	84
Traditional medicine providers	0	6	6
Others (dental services, shops at market, street vendors …)	32	83	115
Total	177 (35.3%)	325 (64.7%)	502 (100%)

Based on the results of the mapping exercise, we then selected a sample of 16 suppliers for in-depth interviews (IDIs) using stratified random sampling that included all identified types of suppliers (including 10 informal suppliers based on the overall proportion for this type of suppliers). The list of the 16 selected suppliers is as follows:

- 1 Public Hospital Pharmacy (formal)
- 1 Commune Health Center (formal)
- 8 Private Dispensaries (4 informal, 4 formal)
- 1 Traditional Medicine Center (informal)
- 2 Private Clinics (informal)
- 3 other types of selling antibiotics (selling at market, community healthcare worker, home visit service) (informal)

Interviews focused on the suppliers’ business and their knowledge of antibiotics and antibiotic resistance. They were asked about the medicines they sold (types, sources, availability, perceived quality, treatment information), their practices associated with antibiotics storage and the dispensing process (choice of antibiotics, length of course, storage and maintenance conditions), and their interactions with customers (customers’ demand, understanding customers’ needs, giving information to customers). They were also asked what they knew about antibiotic resistance and the regulatory aspects for pharmaceutical practices.

In addition, we conducted six focus group discussions (FGDs) and 16 IDIs with adult community members using a purposive sampling approach based on age, gender, those who cared for children ≤5 years old or elderly people > 60, geographic locations, and willingness to participate. The field collaborators identified potential participants who might be willing to speak about their experience on medicines through their local networks and relationships with the community members, and invited them face-to-face or via telephone (no refusal reported from the collaborators).

Six FGDs included four female (one group caring for children aged ≤5 years living in the lowland, one group caring for the elderly in highland, one group aged > 30 in lowland, and one group aged < 30 in mountainous area) and two male groups (one aged < 30 in highland and one aged > 30 in mountainous area). Each FGD consisted of six to nine participants. Of the 16 IDIs, eight included mothers with children ≤5 years old, and eight included participants who did not have children ≤5 years old (two males and two females aged ≥18 to less than 60 years, and two males and two females aged ≥60 years). All participants came from different households, and they were selected following the above sampling framework to ensure a wide range of perspectives in relation to antibiotic access and use in the community. They were asked specifically about their experiences in accessing treatment, their interactions with suppliers, practices associated with antibiotic use, and their general knowledge about the antibiotics and antibiotic resistance. Details on the question guides have been published previously [14].

All IDIs and FGDs were conducted in Vietnamese by three researchers who have experience with the FilaBavi cohort (CNTK, HNH and PHD). These researchers did not live in the study area but they have had established interactions with the community members in other studies, and therefore the participants felt at ease when expressing their opinions during the interviews and discussions. All participants were introduced about the study objectives and procedures, and they signed an informed consent form prior to participating in their IDI or FGD. The IDIs and FGDs took place in a private space at the suppliers’ premises or at the households of the participants (for FGD one household of the participants was used). Each last for 30 min to one hour, and they were digitally recorded. A note-taker wrote down the sequence of each discussion and captured participants’ responses and emotions during the FGDs and IDIs. The sample size of 16 interviews and six FGDs was chosen based on previous experience in qualitative design at the site, and which had provided sufficient data to reach a point of saturation.

### Data analysis

All audio recordings of IDIs and FGDs were transcribed verbatim in Vietnamese and uploaded to Open Code version 4.03 (Umea University, Sweden) for cleaning and analysis. We followed a content analysis approach through a rigorous and systematic process of reading and studying all transcripts and creating and refining codes and categories. Transcripts were read closely to identify initial concepts by one Vietnamese author (HNH), and a preparatory list of themes and categories for coding was generated based on the questions and the identified concepts. Using this list, the transcripts were re-read in detail and coded; each quote was examined to see if it fit within existing codes and any additional inductively derived codes were also included (Table 3). Towards the end of the process, all transcripts were reviewed again, to group responses into broader categories. Results of coding were verified by other Vietnamese authors (PHD and HVTL) for the selected quotes in the analysis. We achieved a high agreement in the initial evaluation (72/78 quotes ~ 92%) among the Vietnamese authors. For the quotes that raised disagreement among the Vietnamese authors, we discussed with all authors and the final decision was made by both HW and JK. The use of both in-depth interviews and focus group discussions in both suppliers and community members facilitated the triangulation of data on antibiotic access and use, therefore contributing to achieve validity, eliminate bias and increase trustworthiness of the findings from our study. Dominant themes and conclusions were drawn on the experiences, perceptions and beliefs of the participants. For each theme reported in this paper, representative quotes were presented to illustrate the experience of the participants; additional quotes can be found in Additional file 1: Table S1. The consolidated criteria for reporting qualitative research (COREQ) guidelines were followed in this paper [16].

**Table 3:** Frequency of responses under the main topics identified from the analysis

Topics	Frequency among study participants		
	Interviews with suppliers (*n* = 16)	Interviews with community (*n* = 16)	Focus group discussions with community (*n* = 49)
Having awareness about antibiotic regulations	13 (81%)		
Customers used previous prescription or others’ prescription	3 (19%)	4 (25%)	6 (12%)
Regulation enforcement is challenging	10 (62%)		
Customers asked for specific antibiotics	7 (44%)	3 (19%)	
Customers returned unused medicines	9 (56%)	1 (6%)	2 (4%)
Drug store as primary healthcare point of contact		9 (56%)	10 (20%)
Recalled common illness that antibiotics were sold/ purchased for (inflammation, sore throat, cold, headache)	13 (81%)	5 (31%)	8 (16%)
Reduce antibiotic sold would affect their business	9 (56%)		
Selling/ purchasing incomplete antibiotic dose	15 (94%)	8 (50%)	7 (14%)
Customers do not complete their antibiotic course	11 (69%)	12 (75%)	17 (35%)
Keep selling unused medicines returned from customers	6 (37%)		1 (2%)
Knowledge: unsure about antibiotics	15 (94%)		
Knowledge: unsure about antibiotic resistance	15 (94%)		
Think that there are many suppliers in the area	6 (37%)	2 (12%)	9 (18%)
Selling antibiotics in a small plastic bag mixed with other medicines	9 (56%)	1 (6%) answered by respondent; 7 (44%) observed by interviewer	
Antibiotics present an important revenue for the suppliers (20–40% total sales)	9 (56%)		
Selling unnecessary antibiotics to increase revenue	4 (25%)		
Sell antibiotics as customer preference	10 (62.5%)	4 (25%)	7 (14%)
Selling antibiotics for animals	2 (12%)		
Selling antibiotics without prescription	15 (94%)	10 (62%)	22 (45%)
Selling unnecessary antibiotics	6 (37%)	2 (12%)	4 (8%)
Information sources for medicines from package inserts or from internet	12 (75%)		
Source of supply for medicines was from pharmaceutical companies (get directly from the companies or delivered by the pharmaceutical representatives)	16 (100%)		
Inappropriate storage condition	8 (50%)		
Taking antibiotic as habit when get sick		6 (37%)	7 (14%)

### Ethical issues

The study protocol was reviewed and approved by the Oxford University Tropical Research Ethics Committee (OxTREC) and the Ethical Committee of Hanoi Medical University (No. 195/HMU IRB).

## Results

Dominant findings emerged from this qualitative analysis are described below including a large number of antibiotic suppliers in the locality, the infringements of regulatory requirements, a reciprocal relationship between suppliers and consumers and the consumer trust on the knowledge and services provided by the suppliers, the habit of using antibiotics in most illness conditions and the confusions about antibiotics in the community. This results section is structured according to these themes.

### A large number of antibiotic suppliers

**Fig. 2:**
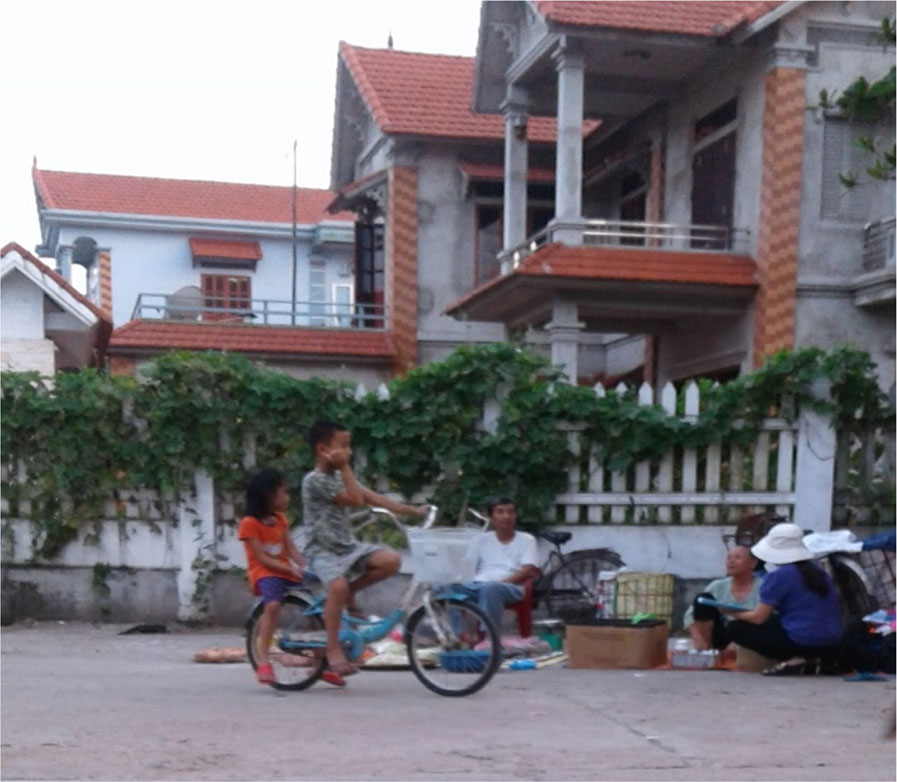
An example of a street vendor. The third woman from the right who sells antibiotics among other drugs in a commune within the FilaBavi site; some drugs were kept in the front plastic box and some products were laid on the cover sheet on the ground

As indicated in our mapping activity, there was a large number of antibiotic suppliers, both formal and informal, operating in the district, with a mean of one supplier per 546 inhabitants. Most suppliers in our IDIs indicated that antibiotics were a significant part of the total number of medicines they had for sale (ranging from 20 to 40%). All suppliers confirmed that customers could get antibiotics from various sources including hospitals, private clinics, pharmacies, drug stores, mobile/ market vendors and traditional healers (Fig. 2). The drug sellers themselves were aware of the high density of medicine suppliers in the area.

“*You see, I am not the only one who sells antibiotic in this area. In this market, there are at least six drug stores so customers have a lot of options depending on convenience and preference”* [unlicensed supplier - private clinic]

Suppliers stated that it was not difficult for them to reach antibiotics from pharmacy companies. They could purchase directly from the companies or ask for door-to-door service through pharmaceutical sales representatives.

*“I always buy antibiotics from a familiar pharmacy company, sometimes from a salesman. At this moment, I have to take care my baby so that the salesman brings medicine to my drug store”* [unlicensed supplier – drug store]

However, the conditions for drug storage were suboptimal in some cases, as indicated in our interviews and observations. There was also a lack of awareness among some suppliers in the requirements for good pharmacy practice, reflected in the expression of some suppliers.

“*We use the air-conditioning in summer only. If the weather is not too hot, I think there is no need to turn it on because the profit from our business cannot afford the electricity bill*”. [licensed supplier - drug store]

*“There is no need to keep the antibiotics in the standard conditions because these drugs are running out quickly”* [unlicensed supplier – traditional medicine store].

*“Antibiotics only need to be stored in a condition protected from light and at room temperature of 30*^*o*^ *C”* [unlicensed supplier – private clinic].

### The rule does not apply here

Despite the legal requirements for suppliers to dispense antibiotics only with prescription, non-compliance is widespread. Fear of losing customers and the need for profit, as well as the inconvenience of getting a prescription are the main reported drivers for this. Both formal and informal suppliers reported that they bypassed the law, without having experienced inspection or fines. The participants also believed the regulations were impractical. Some informal suppliers considered that the regulations on antibiotic prescribing and selling only applied to formal drug stores.

“*I think compliance is impossible to control... There are so many drug suppliers in this area because of the high demand from customers. How could we find enough doctors to prescribe these medications? It is impossible*" [unlicensed supplier – drug store].

*“I think legal suppliers are those who need to follow the law strictly. I do not have any registration so there is no need to follow regulation.”* [unlicensed supplier -private health practitioner]

The perception of “weak antibiotics” was mentioned by some drug suppliers as another justification for their sale of antibiotics. They considered it was legal to sell ‘weak’ antibiotics such as amoxicillin, ampicillin, cephalexin, tetracyclin and chloramphenicol without prescription. These were reported as the best-selling antibiotics locally, and some reported selling these for use in cattle or poultry. Their understanding was that the regulations aimed only to prevent overdispensing of addictive drugs (such as diazepam, codein), or ‘too strong’ antibiotics, not to limit the use of ‘weak’ antibiotics.

*“If someone comes to my store and asks for antibiotics, I usually give them Amo [Amoxicillin] or Cefa [Cefalexin]. After three or five days of taking these, if they do not get better, they need to go to the hospital to get a prescription”* [unlicensed supplier – drug store]

*“The Ministry of Health effectively control prohibited drugs. However, antibiotics are allowed for common use in the community, so people can buy antibiotics easily by themselves”* [unlicensed supplier – drug store].

### A reciprocal relationship and community trust in drug suppliers

To maintain the relationship with their customers, apart from not requiring a prescription before selling antibiotics, suppliers also tend to be responsive to the individual customer’s demands. In almost all cases, suppliers stated that they would sell antibiotics to customers in a quantity less than the recommended dose, and they would accept any unused drugs for refund or to swap for a different drug. It is common practice that, based on the symptoms that customers describe, suppliers provide the customer with a small plastic bag containing different medicines taken out of the original blister strips.

“*I advised them that those medicines should be taken for two days. There was no need to give them the written instruction because they could divide the medicines by themselves … I’m just a pharmacist and I am not permitted to prescribe medicine”* [unlicensed supplier – drug store].

The findings from community IDIs and FGDs were along the same lines. It was common for customers to request specific antibiotics, based on their previous experience. Participants in FGDs also reported that they just took antibiotics for two or three days and stopped once they felt better, reportedly in order to prevent side effects. Antibiotics were also kept for future use for the same symptoms, even though it may not be possible to know the name and expiry dates once pills are taken out of their original packaging. When participants’ health did not improve, they brought the unused drugs back to the store and asked for reimbursement or different medicines. This practice was actually encouraged by some suppliers.

“*Sometimes suppliers indicate to use antibiotics for three days, if there is no effect after two days, I can bring the unused drug back and change for another one”* [FGD - women having children under five].

*“The blister packs were often cut into small pieces for dose dividing so I did not know the expiry dates … if after two days I did not get better I would go to have an examination”* [IDI – male aged 40].

Drug stores are in general the first point of contact for treatment in case of minor illness for community members as they are easily accessible. Most participants indicated that getting medicine from drug stores is the first choice for when they or their children get sick. They are less time consuming, less expensive and overall more convenient than the public health services. Participants also expressed their trust in the local drug suppliers.

*“When I get a mild illness, I always go to drug store. The seller will give me some medicine without any prescription. I don’t even know the name and their effect because I trust them completely. I only go to hospital when I get a severe illness”* [FGD - women having children under five].

*“I believe that the antibiotic supplier is well trained in pharmacy. If I tell him about my problem, I’m sure he will know which drug is right for me.”* [FGD with men over 18 and under 30]

### Habit of using antibiotics driven by both suppliers and customers

It is common for drug suppliers to recommend and sell antibiotics to customers for conditions that do not require antibiotics. This occurs due to lack of knowledge of the suppliers. Customers also appear to play a role in demanding antibiotics when these were not needed. One common example is the use of antibiotics for viral infections.

*“Sometimes I sold antibiotics for diseases that don’t require antibiotics. For example, to a customer with chicken pox, I said she didn’t need to take antibiotics but she still wanted to buy them. So I sold her antibiotics because she wanted it”* [licensed supplier - retail pharmacy].

*“If they have cough or cold I will give them one blister pack of antibiotics: Amoxicilin or Cephalexin, one blister pack of anti-inflammatory drugs, one blister pack of anti-cold drugs and one blister pack of anti-cough drugs.”* [unlicensed supplier – drug store]

Misuse of antibiotics was exacerbated by the lack of knowledge among community members about common conditions, and taking antibiotics has become a habit when they get sick. Some also keep antibiotics at home in case their children get sick. Some individuals stated they were given antibiotics for most illnesses they presented with.

*“Last time, I had a broken toe and the doctor recommended antibiotics for me. Another time I had sore eyes, and the doctor also gave me antibiotics. So most of times when I am sick, I am given antibiotics by health workers”* [FGD – male aged 18-30]*.*

*“When I have a cold, fever and headache, the private doctor often sells six pills of Cephalexin and two for reducing fever and pain … Some doctors only sell the amount enough for two days, if symptoms don’t reduce they’ll change to another drug for the next two days, and if I’m still not recovered they’ll give me injections … Each antibiotic is for a different illness … Cephalexin for cough and sore throat, Ampicillin for diarrhoea”* [IDI – female aged 33].

### Knowledge about antibiotics and antibiotic resistance

There was uncertainty among drug suppliers when asked about how antibiotics work and what antibiotic resistance means. Misperceptions about antibiotics for use against viral infection are common among suppliers. It is also a common perception among drug suppliers that antibiotics are drugs to treat common upper respiratory infections. Most suppliers stated that the major sources of their knowledge about medicines were from package inserts of the drugs or from the internet.

All suppliers had heard about antibiotic resistance but most were unsure what it is and how it develops. Some suppliers correctly identified it as failure of an antibiotic in treating a specific disease, consequently requiring stronger types of antibiotics. Some others perceived it, somewhat vaguely, as a severe outcome of not completing a full course of antibiotics or not following a prescription, while some others confused antibiotic resistance with adverse effects of antibiotics.

From the community side, many participants in community IDIs and FGDs could only recall some general conditions that antibiotics were used for conditions such as inflammation, sore throat, cold and headache. One participant thought antibiotics are medications for ‘most’ diseases. Antibiotics were often identified by color and shape such as red and green capsules or pink long pills. Some confused antibiotics with antipyretics (paracetamol) and anti-inflammatories when they were shown pictures of common drugs in the community.

*“Antibiotics are anti-inflammatory and anti-infective. If you have a cough, antibiotics will make you recover more quickly … In cases of leg pain or cuts in the hands, take antibiotics to prevent infection”* [IDI – female aged 32].

*“Antibiotics are to fight against inflammation (sore throat, blisters) … there is some component of antibiotics in the Panadol [one brand name of Paracetamol] for children as well”* [IDI – female aged 25].

Community members were not aware that antibiotics should be finished with a full-course and that antibiotics can be harmful to their body. The information community members had on antibiotics was usually from previous experience of use, from healthcare workers, from mass media or internet, and, to some extent, from their family members and friends.

## Discussion

We conducted a qualitative study to understand antibiotic access and use in a rural area in northern Viet Nam with the aim of gaining insight into both formal and informal sources of antibiotics used in the community. Widescale non-compliance with, and unawareness of, pharmacy requirements are evident from the suppliers interviewed in our study, both formal and informal. This poses a great challenge for interventions to improve antibiotic access and use in the community. Inappropriate antibiotic use in the community has been highlighted previously in several studies in similar settings [8, 10, 17]. A previous study also reported no difference in the compliance with the prescription-only regulation between pharmacies with and without a Good Pharmacy Practice certificate in Vietnam [8]. Here, we confirmed that antibiotics were reported to be easily obtained at any formal and informal supplier including drug stores, private clinics or street or market vendors, without the need for a prescription as stipulated in the national Drug Law (Table 1). The consistency of the message between interviews with suppliers and community members, as determined through triangulation, suggests that the results are trustworthy.

There appears to be a close, reciprocal and trusting relationship established between suppliers and community members. Antibiotics are easily accessed to the point where they meet the community treatment demands as well as the suppliers’ needs for making a profit. As a result, suppliers continue to sell antibiotics even when aware of the regulations, or that they are selling them for conditions that do not in fact need antibiotic treatment. From the community side, a previous study reported 85% of caregivers of children aged 6–60 months stated that antibiotics were not required if children had a cough without fever [10]. However, still a large proportion of children (71%) took antibiotics during their last mild ARI episode, indicating that factors other than knowledge also influence the decision to use antibiotics. In our study, community members associated antibiotics with severe diseases but mentioned common conditions such as cold, cough, sore throat and headache in their experience with antibiotics. Drug stores were the first point of contact for a number of community members, especially for mild diseases, and as a result their antibiotic use was likely to be influenced by the recommendations of drug suppliers. This was also indicated in a previous study, in which carers of children with symptoms of mild ARI were more likely to seek care at the drug store (38%), and 61% of them were recommended antibiotics [10].

Our study also identified a gap between the drug suppliers’ knowledge and practices in dispensing antibiotics for certain conditions. Previous studies in the same geographical area that assessed the knowledge and practices of private pharmacists and healthcare providers in the management of childhood ARIs also highlighted differences between their knowledge and practices about antibiotics [18, 19]. Nevertheless, community members place a high level of trust on both healthcare professionals and drug suppliers, as reported by participants in our study. The community assumed that drug suppliers should have had adequate qualifications to be able to operate the drug store and dispense drugs. By regulation, staff working at drug stores should be responsible for providing relevant health information to the clients. However, this assumption might be failing given that a large proportion of drug suppliers in this community operates informally without any form of registration from the authorities. In her study, Nga and associates also reported that no pharmacist was present at the observed pharmacies in the rural setting (all of these pharmacies were registered with the authorities) [8]. The issue is not only seen in Vietnam. Despite being considered as an important component in the health system, the performance of pharmacies including formal and informal drug stores has been poor in other LMICs [13]. However, as this review has indicated, interventions should not solely focus on improving knowledge to address the issues of pharmacy practice in Asian settings, but they should also consider the regulatory environment and incentive structures [13].

At the same time, campaigns are necessary to target drug suppliers, both licensed and unlicensed, as well as community members in order to raise awareness about the health implications of inappropriate antibiotic use and consequent antibiotic resistance. The messages should be framed in an easy-to-understand format, with content on the key health conditions where antibiotics are often misused, such as common colds, upper respiratory tract infections or diarrheal diseases. There is evidence of successful public campaigns to improve community antibiotic use in high-income countries [20]. Such campaigns were not devised on an epidemiological and microbiological basis alone, but they also employed behavioural science and social marketing principles and techniques [21]. In Vietnam, some initial evidence shows promising results. A previous study in Hanoi tested a combination of interventions at private licensed pharmacies and reported some improvement in practice after three components were sequentially implemented: regulation enforcement (inspection visits), face-to-face education, and peer influence (group activity) [22]. Another community trial evaluated the effectiveness of an educational package including training on appropriate antibiotic use, case scenario discussion, and poster distribution over a period of seven months for medical and pharmacy personnel in 32 communes in Ba Vi district [23]. Training of staff from private pharmacies has also been reported to improve pharmacy knowledge and practices in other pre- and post- interventions in Vietnam, among which the importance of law enforcement was also highlighted as a means of reducing the gap between staff knowledge and practice [24–26]. These studies did not, however, evaluate the costs of the interventions or how to effectively sustain the effect over the longer term.

The strengths and limitations of our study need to be discussed. A key strength was the sampling approach based on a mapping of all drug suppliers in the area, which allowed us to capture a full spectrum of perspectives in the antibiotic supply network in the community, including from both licensed and unlicensed suppliers. Although our stratified sampling resulted in some types of suppliers having a small number of establishments participating (e.g. only one public hospital pharmacy and one commune health center), the information we collected covers all types of the suppliers that the community members are exposed to in their environment. Our purposive sampling strategy for the community-based FGDs was led by local field collaborators who understand the field context and who could therefore identify knowledge-rich individuals to join the study. With this choice of sampling, we attained depth and richness in the information we collected. Since we have sought to understand antibiotic access and use aspects from the standpoints of both drug suppliers and community members, the findings presented in this paper are likely to reflect many of the processes underlying the antibiotic access and usage in this community.

Our study was conducted in only one rural district of Hanoi and the findings cannot necessarily be generalized to the whole country. However, since Ba Vi district shares typical conditions of socio-economic and health status with many other rural settings in the northern part of Vietnam, it is likely that elements of the results could be applicable elsewhere.

We planned to conduct FGDs among antibiotic suppliers, however, this could not be implemented because the sellers and shop owners would not agree to discuss their knowledge or business practices freely in a shared environment. Nevertheless, information gained from the in-depth individual interviews with the suppliers was rich and reached a point of saturation since no new information emerged during the last interviews conducted. To some extent, the difficulty in organizing FGDs in suppliers reflects a high level of sensitivity towards this issue and somehow indicates the challenges for interventions in targeting the supply channels.

In conclusion, this study sheds light on the practices of antibiotic access and use in a rural setting in Vietnam; it highlights the interactions between antibiotic suppliers and users in the community and identifies possible targets for interventions. These findings can help the government, policy makers and public health professionals to identify appropriate intervention strategies to improve antibiotic access and use in the community. Important strategies that might be effective are law enforcement, continuous medical education of pharmacy personnel, and behavior change campaigns that utilise social marketing techniques and target the wider community. In addition, further research is also required to evaluate the economic implications of such interventions for sustainable outcomes.

## Supplementary information

**Additional file 1: Table S1.** Representative quotes for each theme reported in the results of the qualitative study on antibiotic access and use in FilaBavi.

## Data Availability

De-identified transcripts from interviews and focus group discussions are available on request from the corresponding author.

## Abbreviations

ARI: Acute respiratory infection
FGD: Focus group discussion
IDI: In-depth interview
LMIC: Low and Middle Income Country
