# Supplementary material for: “I can make more from selling medicine when breaking the rules” – understanding the antibiotic supply network in a rural community in Viet Nam
Source: BMC Public Health. 2019 Nov 26;19:1560. doi: 10.1186/s12889-019-7812-z (PMC6880519; doi:10.1186/s12889-019-7812-z)
Supplement: Supplementary file 1 — Additional file 1: Table S1. Representative quotes for each theme reported in the results of the qualitative study on antibiotic access and use in FilaBavi. [file 12889_2019_7812_MOESM1_ESM.docx]

**Supplementary Table 1**

Representative quotes for each theme reported in the results of the qualitative study on antibiotic access and use in FilaBavi

| Theme | Representative quotes |
| --- | --- |
| The unwieldy network of antibiotic suppliers | “*You see, I am not the only one who sells antibiotics in this area. In this market, there are at least 6 drug stores, so customers have a lot of options depending on convenience and preference”* [informal supplier - private clinic]  *“There is no need to keep the antibiotics in the required conditions because these drugs are running out quickly”* [informal supplier – traditional medicine store].  *“Antibiotics only need to be protected from light and stored at room temperature of 30 °C”* [informal supplier – private clinic] |
| The rule does not apply here | *“In general, customers come to my store and ask for antibiotics. If we don’t accept their request, they will go to another store because this village is full of suppliers. If we follow the rules or ask them too thoroughly before giving them medicine, they will not come back* [informal supplier - private health practitioner].  *“To get a prescription, patients need to go to the hospital and get drugs at the hospital pharmacies. If we follow these rules, our business will go bankrupt”* [formal supplier – drug store].  *“Most customers buy antibiotics without any prescription. If they have a prescription, they will buy the drugs at the hospital,and will not come to this small drug store… Customers who are familiar with specific drugs would ask for this drug from the drug seller”* [informal supplier - drug store].  “*I think* *compliance is impossible to enforce. If I’d follow the rules strictly, my drug store will be closed immediately. Moreover, patients would be exhausted while waiting in hospital to get prescription. There are so many drug suppliers in this area because of the high demand from customers. How could we find enough doctors to prescribe these medications? It is impossible*" [informal supplier – drug store].  *“If I follow the rules strictly – selling antibiotics with prescription only - it will be a big problem to my business because the clients coming to buy antibiotics with prescription are very rare”* [informal supplier – drug store].  *“I think legal suppliers are those who need to follow the law strictly. I do not have any registration so there is no need to follow regulation.”* [informal supplier -private health practitioner]  *“Amo [Amoxicillin], Ampi [Ampicillin], Cefa [Cefalexin] are common antibiotics I often sell for customers. They are not as strong as Cefixime and Cefuroxime. Only strong antibiotics need prescription”* [informal supplier – drug store].  *“If someone comes to my store and asks for antibiotics, I usually give them Amo [Amoxicillin] or Cefa [Cefalexin]. After three or five days of taking these, if they do not get better, they need to go to the hospital to get a prescription”* [unlicensed supplier – drug store]  *“The Ministry of Health effectively control prohibited drugs. However, antibiotics are allowed for common use in the community, so people can buy antibiotics easily by themselves”* [unlicensed supplier – drug store]. |
| A reciprocal relationship and community’s trust on drug suppliers | *“I always suggest that they should take the full course of antibiotics (5 – 7 days). However, most of them cannot afford to buy the whole course so they just buy antibiotics for three days or even one. Some customers buy antibiotics for 5 days, but they stop taking the medicine immediately once they feel better. Sometimes they bring the unused drug to my store for refund. If I do not follow their wishes, they will go to another drug store and never come back to me”* [informal supplier -health service at home].  “*The drug stores in a nearby town are still selling antibiotics for the wrong indication, and according to customers’ demand. From the side of customer, people think they understand the disease and use medicine without examination. From the other side, the providers want to make more money*” [formal supplier]  *“Some drug sellers, including myself, sold incomplete courses of antibiotics if the customer asked for it. Sometimes the customer didn’t have enough money to buy the full course. However, some customers said antibiotics were harmful to the body so they stop taking drugs right after they get better”* [informal drug store].  “*I advised them that those medicines should be taken for 2 days. There was no need to give them the written instruction because they could divide the medicines by themselves… you know, I’m just a pharmacist and I am not permitted to prescribe medicine”* [informal supplier – drug store].  “*It’s easy for us to buy antibiotics in this area. When I have a cough, I just come to a drug store near my house and ask for one blister pack of Amo [Amoxicillin], because last time when I had the same symptoms of illness, the seller gave me this kind of medicine*” [a participant of FGD with women between 18 and 30 years of age].  “*70% of mothers having children decide to go to drug stores when their children get sick so we do not have to go to the commune health station. When we go to drug stores we do not have to pay for the examination”* [FGD - females aged over 30].  “*When my daughter gets sick, I always take her to the drug store near my house. After asking about her symptoms, the doctor gives us a bag of medicine. He said these drugs were for 5 days and then I divided the doses myself. I didn’t know what these drugs were and I also didn’t ask the doctor (sic) because I believe him completely. After few days, my child recovered. Although I can get free drugs at the commune health center, I don’t want to bring my child there because last time when she had a cough, the medicine I got did not help her.”* [FGD with mothers having children under 5]  *“I believe that the antibiotic supplier is well trained in pharmacy. If I tell him about my problem, I’m sure he will know which drug is right for me.”* [FGD with men over 18 and under 30]  *“I have a lot of bad experiences at public hospitals. Time consuming, money under the table* [i.e. bribes]*… It even took me a day waiting for results of tests. So, I prefer to go to private clinic”* [FGD - females having children under 5].  *“At public health facilities they always give me medicines of bad quality that can not treat my illness”* [FGD – male aged over 30].  *“The “doctor” mixed all pills in one bag, which was to be divided in 3 dosages and taken in one day. I trusted the doctor’s knowledge, however, I usually do not follow the instructions. I often try to take less pills than prescribed because drugs are not good for the stomach”* [IDI – male aged 40]. |
| Habit of using antibiotics driven by both suppliers and customers | *“Sometimes I sold antibiotics for diseases that don’t require antibiotics. For example, to a customer with chicken pox, I said she didn’t need to take antibiotics but she still wanted to buy them. So I sold her antibiotics because she wanted it”* [licensed retail pharmacy].  *“…used antibiotics for many diseases, including cough and runny nose. Some people they use antibiotics, adding antibiotics to make it go away faster”* [unlicensed drug store].  *“If they have cough or cold I will give them one blister pack of antibiotics: Amoxicilin or Cephalexin, one blister pack of anti-inflammatory drugs, one blister pack of anti-cold drugs and one blister pack of anti-cough drugs.”* [unlisenced supplier]  “*I usually sell Ampicillin and Cephalexin. Cephalexin is used for cold and viral fever… If customers have stomach ache, I will give them both antibiotics and Omeprazole*” [unlicensed supplier – market vendor].  *“When patients come to me with symptoms of chicken pox, I usually give them antibiotics. I know this disease should not be treated with antibiotics but I still sell it to them”* [licensed supplier].  Community:  “*Sometimes suppliers indicate to use antibiotics for 3 days, if there is no effect after 2 days, I can bring the unused drug back and change for another one”* [FGD with mothers that have children under 5]  *“The drug sellers provide verbal instructions and write the instructions on the blister pack. The blister pack were often cut into small pieces for dose dividing so I did not know the expiry dates… if after 2 days I did not get better I would go to have an examination”* [IDI – male aged 40].  *“Last time, I had a broken toe and the doctor recommended antibiotics for me. Another time I had sore eyes, and the doctor also gave me antibiotics. So most of times when I am sick, I am given antibiotics by health workers”* [FGD – male aged 18-30]*.*  *“Everytime when I get a cold, I always take some pills named Dexa [Dexamethasone] and Cefa [Cephalexin] from the drug store. After a few days, I feel really better”* [FGD -females aged over 30 years].  *“Almost any time he had illness or fever or cough I went to the drug store first. Only when taking drugs did not reduce the symptoms we would go to hospital. We even bought drugs to store at home in case he got sick or fever”* [FGD – females aged 18-30 years].  *“When I have a cold, fever and headache, the private doctor often sells 6 pills of Cephalexin and 2 antipyretic pills/ pain killers… they usually give verbal instructions only. Some doctors only sell the amount enough for 2 days, if symptoms don’t reduce they’ll change to another drug for the next 2 days, and if I’m still not recovered they’ll give me injections… Each antibiotic is for a different illness… Cephalexin for cough and sore throat, Ampicillin for diarrhoea”* [IDI – female aged 33]*.*  *“When I get a mild illkness, I always go to drug store. The seller will give me some medicine without any prescription. I don’t even know the name and their effect because I trust them completely. I only go to hospital when I get a severe illness”* [FGD - females having children under 5]. |
| Knowledge about antibiotics and antibiotic resistance | *“Antibiotics are medicine to treat bacteria and inflammation. Sometimes antibiotics are used to kill viruses but the virus will not die completely”* [informal drug store].  *“Antibiotics are synthesized to work against inflammation and infection of the respiratory tract…”* [informal supplier]  *“Antibiotics are medicines that work against virus and bacteria”* [informal mobile health clinic].  *“Antibiotics are medicines for treating some specific diseases such as sore throat, cough, pneumonia, arthritis, injury”* [informal supplier]  *“The most common diseases in this area are cough and cold. It is about 60 – 70 % of total customers who come to my store… I think if patients take antibiotics, they will recover quicker”* [formal supplier].  *“I get information from Google. Some are believable, some I think we should not believe”* [ informal supplier – drug store].  *“I mainly get information from the internet, or from the district health unit about the drugs that they prohibit every now and then they send the notice to us”* [formal supplier – GPP certified drug store].  About antibiotic resistance:  *“Every kind of antibiotic causes different complications. It’s difficult for me to answer what antibiotic resistance is… I think antibiotic resistance means that when you take antibiotics, your body will get some bad effects such as itch, allergy… It is quite dangerous if there is no intervention”* [informal supplier -former healthcare provider].  *“If the antibiotic course is not completed properly, next time antibiotics will not be able to kill the germs. It will cause another disease and next time that antibiotic will not work. Then the antibiotics need to be changed and stronger antibiotics are needed.”* [informal supplier]  *“Antibiotic resistance is extremely dangerous. The disease can be curable but it will take longer than usual.”* [formal supplier]  *“Each physician applied a different treatment method. Following their prescription is one of the best way for me to know how to treat diseases. As far as I know, if someone takes Cefpo (cefpodoxime) or Zinnat (cefuroxime), they couldn’t take Ampicillin any more because Ampicillin is not as “strong” as the antibiotics above”* [informal supplier, private health practitioner]  *“Antibiotic resistance is a phenomenon that you develop an itch or allergy to the medicine. If a patient is not taken to the hospital immediately, it will be serious”* [informal supplier, private health practitioner].  From community members:  *“Antibiotics are anti-inflammatory, anti-infective, if having cough antibiotics will make you recover more quickly… In cases of leg pain or cuts in the hands, take antibiotics to prevent infection”* [IDI – female aged 32]. *“Antibiotics are medicines to kill viruses and bacteria and to increase patient’s immunity”* [FGD – male aged 18-30].  *“Antibiotics are used for treating severe diseases, and used to treat many diseases such as sore throat, common cold, headache. For example, antibiotics are needed for sore throat, otherwise it will not recover”* [IDI – female aged 24].  *“Antibiotics are to fight against inflammation (sore throat, blisters)… there is some component of antibiotics in the antipyretic medicine for children as well”* [IDI – female aged 25].  *“Antibiotics are medicines to treat inflammatory and swelling conditions, many diseases. I used antibiotics 3 to 4 times out of ten times going to have an examination. It has good effects but also bad effects, good is to cure the disease, bad is when using too much that will affect liver and kidney”* [IDI – female aged 65].  “*Antibiotics make the body stronger to kill viruses. When I have an illness or motorbike accident, the doctor also gives me antibiotics, so I think antibiotics are medication for most of diseases*” [FGD – males aged under 30 years].  *“Antibiotics are distinguished by color, shape such as red, green capsule”* [FGD – females having children under 5].  *“I have heard about antibiotics, but I don’t know what antibiotics are. Everytime after visiting the doctor and getting medicine (indicated by the doctor) in the store for my child, I have just given that to my child. During the next time of examination, if the doctor asked me what medicine had been used for my child, I did not know how to answer, I only said that the last time we had used small pink long pills.”* [IDI with a male over 60 years old]  *“I usually check information about antibiotics on the internet. However, I still have the greatest trust in the information the doctor gives me”* [IDI – female aged 33, with one 3-year-old child].  *“I only trust the quality of drugs from the drug store where I usually buy the drugs or the hospital’s pharmacy… I look for information about the drugs from this drug store and I believe it is a reliable source”* [IDI – female aged 22 with 2-year-old child].  *“Sometimes I ask someone having the same problems as me about the drug they use. I think it is one of the useful ways for updating knowledge”* [IDI – female aged over 60]. |
|  |  |
